# Prognostic Factors and Effect of Adjuvant Chemoradiation Following Chemotherapy in Resected Pancreatic Cancer Patients With Lymph Node Metastasis or R1 Resection

**DOI:** 10.3389/fonc.2021.660215

**Published:** 2021-09-22

**Authors:** Jiazhang Xing, Bo Yang, Xiaorong Hou, Ning Jia, Xiaolei Gong, Xiaoyuan Li, Na Zhou, Yuejuan Cheng, Chunmei Bai

**Affiliations:** ^1^Department of Medical Oncology, Peking Union Medical College Hospital, Chinese Academy of Medical Sciences, Beijing, China; ^2^Department of Radiotherapy, Peking Union Medical College Hospital, Chinese Academy of Medical Sciences, Beijing, China

**Keywords:** adjuvant chemotherapy, adjuvant chemoradiation, pancreatic ductal adenocarcinoma, lymph node metastasis, R1 resection

## Abstract

Pancreatic ductal adenocarcinoma (PDAC) is a lethal disease with a poor prognosis. In resectable PDAC, the recurrence rate is still high even when surgery and adjuvant chemotherapy (CT) are applied. Regional lymph node metastasis and positive margins are associated with higher recurrence risk and worse survival. Adjuvant radiotherapy has been explored, but its efficacy remains controversial. In recent years, some characteristics have been reported to stratify patients who may benefit from adjuvant chemoradiation (CRT), such as lymph node metastasis and margin status. Adjuvant chemotherapy followed by chemoradiation (CT-CRT) was also proposed. A total of 266 patients with resectable PDAC who have lymph node metastasis or R1 resection after surgery were enrolled. In multivariate Cox regression analyses, pancreatic body or tail tumor location (HR 0.433, p<0.0001, compared with pancreatic head) and adjuvant CT predicted a better survival, while there were no significant differences among the different CT regimens. Higher T stage indicated poor survival (stage I: reference; stage II: HR 2.178, p=0.014; stage III: HR 3.581, p=0.001). Propensity score matching was applied in 122 patients to explore the role of CRT. A cohort of 51 patients (31 and 20 patients in the CT and CT-CRT groups, respectively) was generated by matching. Further analyses revealed adjuvant CT-CRT was associated with prolonged survival compared with CT alone (HR 0.284, p=0.014) and less frequent local recurrences (56.5% *vs*. 21.4% in the CT and CT-CRT group, respectively). However, no significant differences in disease-free survival among these two groups were observed.

## Introduction

Pancreatic ductal adenocarcinoma (PDAC) is highly malignant with a dismal prognosis. Although surgery is the only potentially curative method, up to 80% of patients who undergo curative resection experience recurrence within two years, and the 5-year survival rate is 10-25% (1, 2). Lymph node metastasis and positive margins indicate a poor prognosis. Lymph node metastasis is associated with worse disease-free survival (DFS) and overall survival (OS) (3, 4). R1 resection is also associated with decreased OS (5). Adjuvant chemotherapy (CT) can improve long-term outcomes compared with observation. Based on the PRODIGE-24 and ESPAC-4 trials (6, 7), six months of mFOLFIRINOX or gemcitabine with capecitabine is the recommended adjuvant CT regimen. Gemcitabine monotherapy is an alternative option for patients who cannot tolerate combination therapy. In the Asian population, S-1 was shown to markedly prolong OS with lower toxicity than gemcitabine (8). However, after receiving surgical treatment and standard adjuvant CT, patients are still at high risk of local relapse (6). Therefore, adjuvant radiotherapy has been investigated for many years in the treatment of resectable pancreatic adenocarcinoma.

Due to the lack of high-quality clinical trials, the role of radiotherapy is still controversial. Adjuvant chemoradiation (CRT) alone did not show any OS benefit over observation due to the toxicity of radiotherapy and the delay of the administration of CT (9–11). Therefore, some researchers have investigated the role of adjuvant chemotherapy and chemoradiation (CT-CRT). However, for non-selected patients, no differences in DFS and OS were observed between adding CRT to adjuvant CT and administering CT alone according to several phase 2 and phase 3 trials (12, 13). Patients with certain pathological characteristics may benefit from additional CRT. Lymph node metastasis favors improved OS from CT-CRT when compared to CT or CRT alone (0 positive nodes: hazard ratio (HR) 0.96, P = 0.67; 1‐3 positive nodes: HR 0.74, P < 0.001; 4+ positive nodes: HR 0.75, P < 0.001) (14). Positive resection margin is also an indicator of survival benefit from CRT (15). Therefore, the American Society of Clinical Oncology (ASCO) guidelines (16) recommend that adjuvant CRT may be offered to patients with R1 resection or node-positive disease after 4 to 6 months of systemic adjuvant CT. However, the view of the role of CT-CRT is not consistent among different international academic societies, and the European Society for Medical Oncology (ESMO) guidelines do not recommend adjuvant CT-CRT (17).

Our study enrolled 266 patients with R1 resection or node-positive pancreatic cancer after surgery. Survival analysis based on baseline characteristics and treatment was performed. Propensity score matching (PSM) was used to investigate the role of radiotherapy in 122 patients who had medical records of adjuvant therapy.

## Methods

### Patients

This study was reviewed and approved by the ethics committee of Peking Union Medical College Hospital (PUMCH). The study sample comprised consecutive patients who received a pathological diagnosis of pancreatic adenocarcinoma and underwent curative-intent surgery between 1 January 2015 and 1 October 2019 at PUMCH. All enrolled patients had lymph node metastasis or R1 resection. R1 resection was defined as a positive margin within less than 1 mm according to the 8^th^ American Joint Committee on Cancer (AJCC) manual (18).

The exclusion criteria were a history of other cancers, R2 resection and distant metastasis before surgery. Pathologic characteristics, including tumor location, tumor size, lymph node metastasis and margin status, were collected from the original pathology reports. Clinical characteristics, including sex, age at diagnosis, CA19-9 before CT, CT information, radiotherapy information and follow-up records, were obtained from detailed clinical records. All the patients were restaged pathologically according to the 8th AJCC TNM classification (18). The follow-up duration was from the surgery date to 1 October 2020.

Patients with CA19-9 levels lower than three times the upper limit of normal before CT and having adjuvant treatment records in the hospital were selected to perform further PSM analysis. The patients were categorized into two groups: the CT group and the CT-CRT group. The CT-CRT group only enrolled patients who did not have distant metastasis before radiotherapy. First-recurrence sites were analyzed in the after-matching cohort. By radiologic records, local recurrence was classified as recurrences in the remnant pancreas, the surgical bed, or locoregional nodes. Other recurrences outside these areas were defined as distant recurrence (19). The DFS was calculated as the time from surgery to the first event of recurrent disease, death, or last follow-up.

### Statistical Analysis

Statistical analyses were performed using SPSS version 26.0 (IBM Corp., NY). Continuous variables are summarized as median values with interquartile range, and categorical outcome variables are summarized as frequencies with percentages. The Kaplan-Meier method was used to investigate survival in different treatment modalities, CT regimens, pathological T and N staging and TNM stage groups. The Cox proportional hazards method was used to select significant independent predictors for survival. Multiple linear regression was applied to analyze the correlation of variables in the Cox regression model. Only factors with a p-value less than 0.1 in the univariate Cox regression analyses were included in further multivariate Cox regression analyses.

The PSM method was used to balance the potential confounding factors between the CT and CT-CRT groups. A multivariable logistic regression model was used to create propensity scores that included the following covariates: age, sex, tumor location, margin status, pathological T and N staging, TNM stage, pre-CT CA19-9, CT regimen and cumulative dose. A caliper of 0.2 propensity score standard deviations was used. Patients in the adjuvant CT-CRT group were matched at a ratio of 1:2 to patients in the adjuvant CT group. Differences in patient characteristics among the matched groups were evaluated using Student’s independent-sample T-test and the chi-square test.

## Results

### Patients

This study enrolled 266 patients with lymph node metastasis or R1 resection after pancreatic adenocarcinoma surgery. The median age at diagnosis was 61.5 years. A total of 148 (55.6%) of the enrolled patients were male. A total of 69.2% of patients had tumors located in the pancreatic head. Ninety-one patients had an R1 resection. Most patients had stage T2 tumor and TNM stage II disease. Regarding lymph node status, few patients (7.5%) did not have lymph node metastasis, while 63.9% of patients had N1 stage and 28.6% of patients had N2 stage. The main CT regimens used were gemcitabine monotherapy (GEM), S-1 monotherapy, and gemcitabine plus oral fluoropyrimidine combination regimen. S-1 was the most commonly used oral fluoropyrimidine drug in our study, while capecitabine was also used. Thirty-seven patients did not receive CT because of intolerance or unwillingness. A total of 9.4% of patients received adjuvant fluoropyrimidine-based CRT (radiation dose: median total dose: 50.0 Gy, range: 45.0-56.0 Gy; median fraction dose: 1.8 Gy, range: 1.8-2.0 Gy) after 2-6 cycles of CT. The baseline characteristics are provided in **Table 1**. Among the 266 patients, 122 patients had adjuvant treatment records including regimen, dose and cycles in our center.

**Table 1:** Baseline characteristics of 266 patients with lymph node metastasis or R1 resection after surgery for resectable pancreatic adenocarcinoma.

Characteristics	N (266)	%
Age, years, median (IQR)	61.5 (55.0-67.0)	
Sex		
Male	148	55.6
Female	118	44.4
Location		
Head	184	69.2
Body/tail	82	30.8
Margin status		
R0	175	65.8
R1	91	34.2
AJCC TNM stage		
Stage I	15	5.6
Stage II	175	65.8
Stage III	76	28.6
AJCC T stage		
T1	47	17.7
T2	167	62.8
T3	52	19.5
AJCC lymph node stage		
N0	20	7.5
N1	170	63.9
N2	76	28.6
Chemotherapy regimen		
None	37	13.9
GEM	64	24.1
GS	82	30.8
S-1	51	19.2
mFOLFIRINOX	4	1.5
Unknown	28	10.5
Radiotherapy		
No	241	90.6
Yes	25	9.4

GEM, gemcitabine monotherapy; GS, gemcitabine plus oral fluoropyrimidine combination regimen.

### OS Analysis

**Figure 1:**
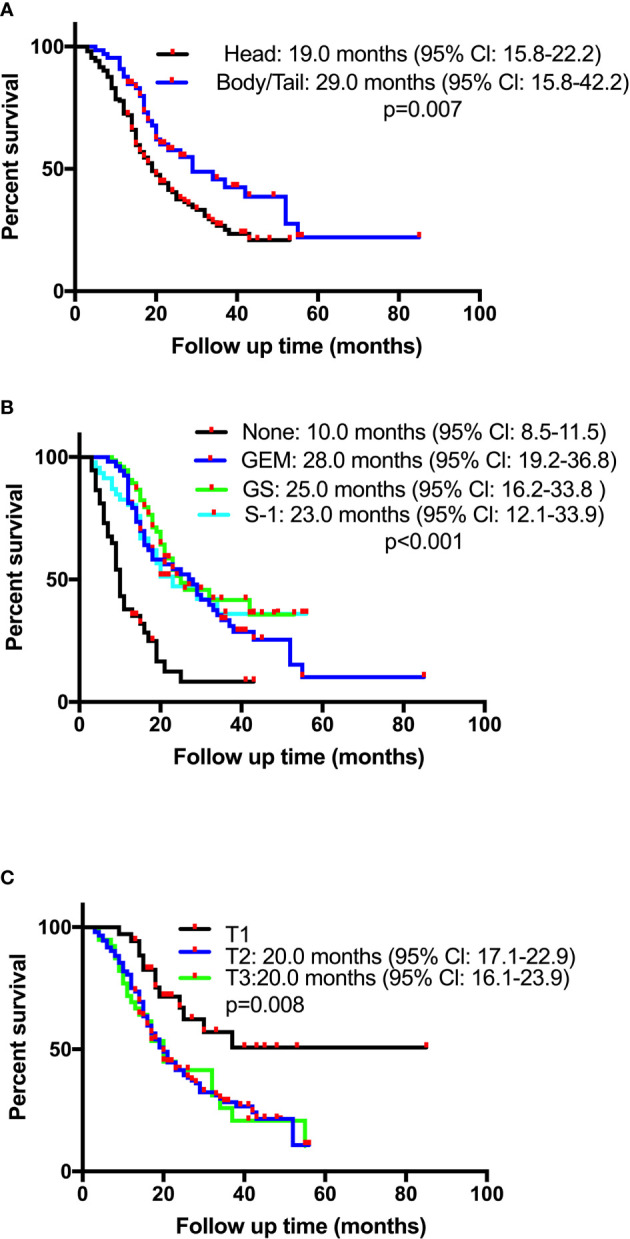
Kaplan-Meier overall survival (OS) curves in patients with resectable pancreatic adenocarcinoma who underwent surgery and had lymph node metastasis or R1 resection based on **(A)** tumor location, **(B)** chemotherapy, or **(C)** pathological T stage. GEM, gemcitabine monotherapy; GS, gemcitabine plus oral fluoropyrimidine combination regimen.

Survival time of the enrolled patients ranged from 3 to 55 months, with a median of 21 months. OS rates at 1, 3, and 5 years of 77.1%, 32.3%, and 15.7%, respectively. The median follow-up time was 18 months (range 3-85), with 218 (82.0%) patients showing a response. The median OS for patients receiving CT-CRT was 52.0 months (95% Cl: 38.5-65.5) compared with 20.0 months for those receiving CT (95% Cl: 17.6-22.4). The median OS for patients with tumors located in the pancreatic head was 19.0 months (95% Cl: 15.8-22.2), while for patients with tumors located in the pancreatic body or tail, it was 29.0 months (95% Cl: 15.8-42.2) (**Figure 1A**). The median OS times for patients who received GEM alone, gemcitabine with S-1 (GS) and S-1 monotherapy were 28.0 months (95% Cl: 19.2-36.8), 25.0 months (95% Cl: 16.2-33.8) and 23.0 months (95% Cl: 12.1-33.9), respectively (**Figure 1B**). Four patients received mFOLFIRINOX therapy, three of whom died, and their survival times were 8.0, 14.0 and 17.0 months. The median OS for patients with stage T2 and T3 tumors was 20.0 months, with 95% Cl values of 17.1-22.3 and 16.1-23.9, respectively. Patients with stage T1 tumors had better survival, with an OS of 37.0 months and a 50.8% cumulative survival rate (**Figure 1C**). According to the univariate Cox regression analyses, seven factors had a p-value less than 0.1 (**Table 2**). By multiple linear regression analysis, there was a correlation between the TNM stage and lymph node stage. Because the T stage and lymph node stage were included, the TNM stage was removed to modified our multivariable Cox regression model. Multivariate Cox regression analyses (**Table 2**) revealed that pancreatic body or tail tumor location (HR 0.434, p<0.0001, compared with pancreatic head), adjuvant CT-CRT (HR 0.369, p=0.015, compared with CT alone) and CT (GEM: HR 0.356, p<0.0001; GS: HR 0.269, p<0.0001; S-1: HR 0.325, p<0.0001, compared with no CT) were significant independent predictors for better OS. Higher T stage indicated poor survival (stage I: reference; stage II: HR 2.199, p=0.013; stage III: HR 3.796, p<0.0001).

**Table 2:** Cox proportional hazards regression analysis of the risk of death among 266 patients with lymph node metastasis or R1 resection after surgery for resectable pancreatic adenocarcinoma.

Characteristics	Univariate analysis			Multivariate anlaysis		
	HR	95%Cl	P value	HR	95%Cl	P value
Age	1.015	0.998-1.033	0.081	1.020	1.000-1.040	0.053
Sex						
Male	Ref					
Female	0.919	0.652-1.294	0.628			
Location						
Head	Ref			Ref		
Body/tail	0.584	0.392-0.871	0.008	0.434	0.275-0.685	<0.0001
Margin status						
R0	Ref					
R1	1.000	0.696-1.436	1.000			
AJCC TNM stage						
Stage I	Ref					
Stage II	2.197	0.890-5.422	0.088			
Stage III	2.663	1.044-6.796	0.040			
AJCC T stage						
T1	Ref			Ref		
T2	2.348	1.313-4.198	0.004	2.199	1.183-4.086	0.013
T3	2.440	1.257-4.733	0.008	3.796	1.856-7.756	<0.0001
AJCC lymph node stage						
N0	Ref			Ref		
N1	1.896	0.871-4.124	0.107	1.513	0.682-3.358	0.309
N2	2.293			1.748	0.753-4.060	0.194
Chemotherapy regimen						
None	Ref					
GEM	0.318	0.196-0.515	<0.0001	0.356	0.223-0.598	<0.0001
GS	0.234	0.143-0.382	<0.0001	0.269	0.160-0.451	<0.0001
S-1	0.286	0.167-0.491	<0.0001	0.325	0.187-0.564	<0.0001
mFOLFIRINOX	0.517	0.158-1.694	0.276	0.690	0.198-2.410	0.561
Radiotherapy						
No	Ref			Ref		
Yes	0.286	0.132-0.618	0.001	0.369	0.165-0.826	0.015

HR, hazard ratio; Cl, confidence interval; Ref, reference.

GEM, gemcitabine monotherapy; GS, gemcitabine plus oral fluoropyrimidine combination regimen.

### PSM

A total of 122 patients with adjuvant treatment records after surgery were categorized into two groups: the CT group (97 patients) and the CT-CRT group (25 patients). The matching procedure was successfully improved balance in age, sex, tumor location, marginal status, AJCC TNM staging, pre-CT CA19-9, CT regimen and cumulative CT dose between the two groups. It yielded a cohort of 51 patients (31 and 20 patients in the CT group and CT-CRT group, respectively) for further analyses (**Table 3**). Because few patients in the CT-CRT group received S-1 and sequential CRT, the CT regimens only included GEM and GS after matching.

**Table 3:** Baseline characteristics before and after propensity score matching.

	Before PSM			Aefore PSM		
Characteristics	CT (n = 97)	CT-CRT (n = 25)	P value	CT (n = 31)	CT-CRT (n = 20)	P value
Age(mean ± SD)	60.7±9.8	60.3 ± 11.8	0.87	57.5 ± 10.8	58.8 ± 11.5	0.69
Sex			0.70			0.97
Male	54	15		20	13	
Female	43	10		11	7	
Location			0.70			0.94
Head	66	16		22	14	
Body/tail	31	9		9	6	
Marginal status			0.05			0.81
R0	70	13		16	11	
R1	27	2		15	9	
AJCC TNM stage			0.38			0.83
Stage I	5	3		2	2	
Stage II	73	16		21	12	
Stage III	19	6		8	6	
AJCC T satge						
T1	14	8	0.12	8	7	0.70
T2	62	13		18	11	
T3	21	4		5	2	
AJCC lymph node stage						
N0	6	4	0.21	2	3	0.52
N1	72	15		21	11	
N2	19	6		8	6	
Pre-chemotherapy CA199						
>37	30	7	0.78	9	5	0.75
≤37	67	18		22	15	
Chemotherapy regimen						
GEM	36	11	0.39	16	10	0.91
GS	37	11		15	10	
S-1	24	3		0	0	
Chemotherapy cumulative dose(mg)						
Gemcitabine(Median, IQR)	21300 (14400)	24900 (19200)	0.13	22800 (16800)	25200 (19200)	0.76
Fluropyrimidine(Median, IQR)	8400 (3780)	11760 (9380)	0.03	9120 (7840)	10920 (9380)	0.93
IMRT dose(Gy)						
Median total dose		50			50	
Median fraction dose		1.8			2	

GEM, gemcitabine monotherapy; GS, gemcitabine plus oral fluoropyrimidine combination regimen.

Thirty-seven patients in the after-matching cohort experienced disease recurrence, 23 patients from the CT group (74.2%) and 14 from the CT-CRT group (70%). 56.5% of recurrences in the CT group were local, 26.1% were distant, and 17.4% were both local and distant. In the CT-CRT group, 21.4% of recurrences were local, 50% were distant, and 28.6% were local and distant. Patients in the CT-CRT group were less likely to have local recurrences than those in the CT group. Median DFS in the CT-CRT group was 19.0 months (95% CI, 15.2 to 22.6 months) versus 14.0 months (95% CI, 9.6 to 18.4 months) in the CT group, while no significant differences among these two groups were observed.

**Figure 2:**
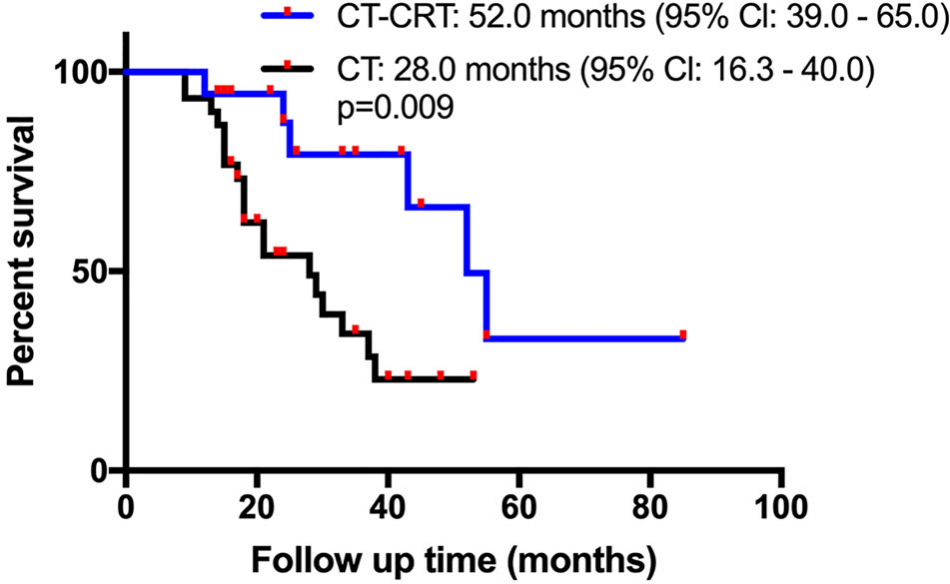
Kaplan-Meier overall survival (OS) curves in patients with resectable pancreatic adenocarcinoma who underwent surgery and had lymph node metastasis or R1 resection after propensity score matching. Adjuvant chemotherapy (CT) *vs* adjuvant chemotherapy and chemoradiation (CT-CRT).

According to the univariate Cox regression analysis, the HR derived for the adjuvant CT-CRT cohort compared with the adjuvant CT alone cohort was 0.284 (0.104–0.778), with a p-value of 0.014, indicating that adjuvant CT-CRT was a significant prognostic risk factor for a more favorable OS. After matching, the median OS time for patients receiving CT-CRT was 52.0 months (95% Cl: 39.0-65.0) compared with 28.0 months for those receiving CT (95% Cl: 16.3-40.0) (**Figure 2**). Because other factors in univariate Cox regression analysis did not show significance, further multivariate Cox regression analyses were not performed.

## Discussion

Patients with lymph node metastasis or R1 resection after surgery had a poor prognosis (3–5). These two factors were also reported as possible indicators for selecting patients who may benefit from adjuvant radiotherapy (14, 15). Therefore, in our study, we focused on these patients to analyze possible prognostic factors and explored the role of postoperative CT-CRT.

In our analyses, pancreatic tumors located in the pancreatic body or tail indicated a better prognosis than tumors located in the pancreatic head for patients undergoing curative resection. Several studies reported similar results (20–22). Pathologically unfavorable is a possible explanation for the worse OS associated with tumors located in the pancreatic head, which tend to have lymph node metastases, advanced pathologic stages and worse tumor grades (21). We also found that the pathological T stage was a significant prognostic factor. Indeed, the significance of tumor size in clinical outcomes has received more attention since the reversion of the T stage in the 8th edition of the AJCC TNM staging system (18). Many studies have validated the more detailed T staging’s superiority in stratifying patients by survival (23, 24). Larger tumors correlated with higher CA19-9 levels, higher grades, perineural invasion, R1 resection and more positive lymph nodes (25). Our study indicated that tumor location and size should be given more attention in patients with R1 resection or positive lymph nodes. The role of tumor location and size in treatment stratification for these high recurrence risk patients could be further explored. In this study, the N and TNM stage were significantly associated with survival in univariate Cox regression analyses but not in multivariate analyses. The few N0 and TNM stage I patients led to the patients’ uneven stage distribution, contributing to the bias. An association between marginal status (R0 *vs* R1) after resection and OS was not found. The criteria we set for patient selection resulted in all patients with R0 resection included in this study having lymph node metastasis, which also indicated a poor prognosis. Thus, the survival difference between different marginal status groups was not revealed. The CT regimen was also recorded and analyzed in our study. For patients after resection of pancreatic adenocarcinoma, mFOLFIRINOX or gemcitabine with capecitabine is the recommended adjuvant CT regimen, although the increased toxicities of mFOLFIRINOX limit its widespread use (7). Gemcitabine monotherapy is an alternative option. In the Asian population, the S-1 regimen was indicated to markedly prolong OS with lower toxicity than gemcitabine (8). In our study, only four patients received the mFOLFIRINOX regimen, and most of the patients included received the GEM, GS or S-1 regimen. CT showed a significant improvement in OS compared with observation, while there were no significant differences among the different CT regimens.

Due to the high risk of pancreatic cancer recurrence after surgery and CT (6), the effectiveness of adjuvant radiotherapy has been explored for many years. However, compared with CT, the role of radiotherapy in the adjuvant setting is controversial. In our study, PSM was applied and generated a cohort for further analysis (31 patients in the CT group and 20 patients in the CT-CRT group). According to the following Cox regression analysis, adjuvant CT-CRT was a significant prognostic factor for a more favorable OS (p=0.014). Some studies also supported the survival benefit of CT-CRT (26). The prolonged OS of the CT-CRT group might benefit from local control improvement. The local recurrence rate of the CT-CRT group was lower than that of the CT group. The improvement of local control by adding CRT in pancreatic cancer therapy was illustrated in other studies. In a phase II prospective randomized study comparing adjuvant CT alone and adjuvant CT-CRT, the local and simultaneous local and distant progression rates were 11% and 13%, respectively, in the CT-CRT group, which were lower than those (24% and 20%) in the CT group (12). Other retrospective studies also reached similar results (26–28). According to previous studies, the risk of local recurrence correlated with lymph node metastasis (19, 29) and R1 resection (30–32). The better outcome in the CT-CRT group in this study, of which patients have lymph node metastasis or R1 resection, might indicate the importance of patients selecting. Patients who have a higher risk of local recurrence were likely to benefit from CT-CRT. Meanwhile, some pathological characteristics have been reported to indicate benefit from adding adjuvant radiotherapy, such as pT3 (33), lymph node metastasis (14, 29, 33, 34) and positive margin status (35).

In the ESPAC 4 study, for patients with a positive margin, gemcitabine combined with capecitabine did not significantly improve patients’ overall survival compared to gemcitabine alone (23.7 *vs*. 23.0 months) (6). Although the mFOLFIRINOX regimen showed higher activity in nodal positive or R1 resection pancreatic cancer, few patients could tolerate the toxicities. Therefore, adjuvant radiotherapy could be taken into consideration in these patients. Our study focused on these patients for the first time to analyze and prove the survival benefit of CT-CRT. According to previous studies, the delayed administration of CT, toxicities of radiotherapy (9–11) and radiation dose were potential reasons for the lack of OS benefit observed in CRT or CT-CRT (36). In our study, all patients received a standard regimen, including CRT following 4-6 months of CT, recommended by the ASCO guidelines (16). Intensity-modulated radiation therapy (IMRT) was used in all patients with a sufficient radiotherapy dose (median total dose of 50 Gy and fraction dose of 1.8 Gy). IMRT has been widely used in recent years to achieve more conformal dose delivery with less toxicity. In pancreatic cancer, IMRT had significantly reduced toxicities without changes in the therapeutic outcome compared with conventional 3D-conformal radiation therapy (37). Thus, our study results could be more convincing by adapting reasonable regimens and advanced technology. For the CT regimen given before adjuvant CRT, there were no differences in DFS and OS between gemcitabine and fluoropyrimidine-based regimens (38, 39). However, a trend to extend OS in the gemcitabine group for the patients with pancreatic adenocarcinoma in the pancreatic head was reported (39). In our study, the number of patients who received GS was equal to those who received gemcitabine alone.

There are some limitations of our study. First, it was a retrospective study based on single center. Second, the small sample size of patients who received CT-CRT limited a definitive conclusion. Third, about half of the patients enrolled in our study received S1 chemotherapy, which markedly prolonged OS with low toxicity in Asian patients but not demonstrated in European and North American patients. Hence, our results should be cautiously illustrated when applied in non-Asian populations.

Further studies are in need to illustrate the survival benefit in selected patients. Biomarkers such as genetic alteration, circulating tumor DNA or circulating tumor cells should also be incorporated to investigate the predictive biomarker for chemoradiation. Phase III trial aimed at offering more convincing evidence for CT-CRT is ongoing, in which patients receive adjuvant CRT [50.4 Gy in 28 fractions with concomitant 5-fluorouracil (5-FU)] after five cycles of adjuvant CT (40).

In conclusion, our study focused on a subgroup of patients with resected pancreatic cancer who had a worse prognosis due to lymph node metastasis or positive margin status. Among the baseline and pathological characteristics, tumor location and the T stage were significantly associated with prognosis. There was no significant difference in the effectiveness of different CT regimens. PSM and Cox regression analysis illustrated that adjuvant CT-CRT was associated with prolonged patient OS and lower local recurrence rate compared with CT alone.

## Data Availability Statement

The original contributions presented in the study are included in the article/supplementary material. Further inquiries can be directed to the corresponding authors.

## Ethics Statement

The studies involving human participants were reviewed and approved by Peking Union Medical College Hospital. The patients/participants provided their written informed consent to participate in this study.

## Author Contributions

JX, YC and CB conceived and designed the study. BY and XH collected the clinical data. NJ, XG, XL and NZ finished patients’ follow-up. JX and YC performed the statistical analyses. JX wrote the manuscript. YC and CB reviewed and revised the manuscript. All authors contributed to the article and approved the submitted version.

## Funding

This study received grants from the Chinese Academy of Medical Sciences (CAMS) Initiative for Innovative Medicine (CAMS- I2M) 2017-I2M-1-001.

## Conflict of Interest

The authors declare that the research was conducted in the absence of any commercial or financial relationships that could be construed as a potential conflict of interest.

## Publisher’s Note

All claims expressed in this article are solely those of the authors and do not necessarily represent those of their affiliated organizations, or those of the publisher, the editors and the reviewers. Any product that may be evaluated in this article, or claim that may be made by its manufacturer, is not guaranteed or endorsed by the publisher.
